# Analysis of cat oocyte activation methods for the generation of feline disease models by nuclear transfer

**DOI:** 10.1186/1477-7827-7-148

**Published:** 2009-12-11

**Authors:** Chunmin Wang, William F Swanson, Jason R Herrick, Kiho Lee, Zoltan Machaty

**Affiliations:** 1 Department of Animal Sciences, Purdue University, West Lafayette, IN 47907, USA; 2 Center for Conservation and Research of Endangered Wildlife, Cincinnati Zoo and Botanical Garden, Cincinnati, OH 45220, USA; 3 College of Veterinary Medicine, University of Illinois at Urbana-Champaign, Urbana, IL 61802, USA

## Abstract

**Background:**

Somatic cell nuclear transfer in cats offers a useful tool for the generation of valuable research models. However, low birth rates after nuclear transfer hamper exploitation of the full potential of the technology. Poor embryo development after activation of the reconstructed oocytes seems to be responsible, at least in part, for the low efficiency. The objective of this study was to characterize the response of cat oocytes to various stimuli in order to fine-tune existing and possibly develop new activation methods for the generation of cat disease models by somatic cell nuclear transfer.

**Methods:**

First, changes in the intracellular free calcium concentration [Ca2+]i in the oocytes induced by a number of artificial stimuli were characterized. The stimuli included electroporation, ethanol, ionomycin, thimerosal, strontium-chloride and sodium (Na+)-free medium. The potential of the most promising treatments (with or without subsequent incubation in the presence of cycloheximide and cytochalasin B) to stimulate oocyte activation and support development of the resultant parthenogenetic embryos was then evaluated. Finally, the most effective methods were selected to activate oocytes reconstructed during nuclear transfer with fibroblasts from mucopolysaccharidosis I- and alpha-mannosidosis-affected cats.

**Results:**

All treatments were able to elicit a [Ca2+]i elevation in the ooplasm with various characteristics. Pronuclear formation and development up to the blastocyst stage was most efficiently triggered by electroporation (60.5 +/- 2.9 and 11.5 +/- 1.7%) and the combined thimerosal/DTT treatment (67.7 +/- 1.8 and 10.6 +/- 1.9%); incubation of the stimulated oocytes with cycloheximide and cytochalasin B had a positive effect on embryo development. When these two methods were used to activate oocytes reconstructed during nuclear transfer, up to 84.9% of the reconstructed oocytes cleaved. When the 2 to 4-cell embryos (a total of 220) were transferred into 19 recipient females, 4 animals became pregnant. All of the fetuses developed from oocytes activated by electroporation followed by cycloheximide and cytochalasin B incubation; no fetal development was detected as a result of thimerosal/DTT activation. Although heartbeats were detected in two of the cloned fetuses, no term development occurred.

**Conclusion:**

Electroporation proved to be the most effective method for the activation of cat oocytes reconstructed by nuclear transfer. The combined thimerosal/DTT treatment followed by cycloheximide and cytochalasin B incubation triggered development effectively to the blastocyst stage; whether it is a viable option to stimulate term development of cloned cat embryos needs further investigations.

## Background

Ovulated mammalian oocytes are arrested at the metaphase stage of their second meiotic division [1]. Normally, they resume meiosis and enter the first interphase at the time of fertilization when the fertilizing sperm activates the oocyte's developmental program by triggering changes in its intracellular free calcium concentration [Ca^2+^]_i_. Changes in the [Ca^2+^]_i _can also be induced artificially and as a result parthenogenetic oocyte activation can take place. Although mammalian parthenogenetic embryos never develop to term, a great number of invertebrate and vertebrate animal species are able to reproduce via parthenogenesis [2].

During parthenogenetic activation, the increase in the [Ca^2+^]_i _must be able to trigger the numerous biological events that are associated with fertilization [3]. The process is an integral part of several assisted reproductive technologies and has particular relevance in somatic cell nuclear transfer [4]. Numerous oocyte activation methods have been designed to mimic the Ca^2+ ^signal induced by the sperm; however, very few of them are able to generate the oscillatory Ca^2+ ^signal seen during mammalian fertilization. Thus in most cases a single [Ca^2+^]_i _rise is induced to stimulate development of the reconstructed oocyte [5]. Although this was shown capable of triggering oocyte activation [6], the amplitude, frequency and duration of repetitive Ca^2+ ^signals are believed to have profound effects not only on the immediate events of oocyte activation but also on peri-implantation development [7].

Activation of oocytes of many domestic species including the domestic cat has been described and used in reproductive research [8]. Cats are useful research models for a number of reasons. They are valuable for the study of hereditary diseases in humans since they can provide insight into disease etiology and pathology [9-13]. Cat models also facilitate investigation of promising treatments including gene therapies, as recently shown for the lysosomal storage diseases mucopolysaccaridosis (MPS) and α-mannosidosis (AMD); [14,15]. Reproductive research on domestic cats is also important for conserving endangered felid species [16-18]. Somatic cells isolated from nondomestic felids can be transferred into enucleated domestic cat oocytes, and it has been demonstrated that this nuclear transfer approach has the potential of generating live offspring if the two felid populations are not too distantly related [19]. Despite the occasional successes low birth rates after nuclear transfer, just like in most other species, remain a formidable challenge. One potential reason for the low efficiency is poor embryo development following activation of the reconstructed oocytes. The number of methods known to induce parthenogenetic development of cat oocytes is rather low; the published methods are limited to electroporation, ethanol and the ionophore A23187 [8,19]. In most cases activation techniques developed for other species have been adopted for producing the cloned cat embryos; this may limit embryonic development after embryo transfer. The aims of this study were to 1) characterize [Ca^2+^]_i _changes in cat oocytes induced by different stimuli; 2) develop new activation methods that could be applied after nuclear transfer to produce cleavage-stage embryos; and 3) assess the feasibility of nuclear transfer to produce cloned cats affected with the lysosomal storage diseases MPS or AMD.

## Methods

All chemicals were purchased from Sigma-Aldrich Chemical Company (St. Louis, MO, U.S.A.) unless otherwise indicated. Experiments were conducted according to guidelines approved by the Institutional Animal Care and Use Committee (IACUC) at the Cincinnati Zoo & Botanical Garden.

### In vitro oocyte maturation

Cat ovaries were collected after routine ovariectomy. Cumulus-oocytes complexes (COCs) were collected by slicing in Hepes-buffered Tyrode's Lactate (TL-Hepes) medium and matured in groups of 10 in 100-μl drops of Feline Optimized Culture Medium (FOCM; [20]) supplemented with 0.1 mM cysteamine, 0.6 mM cysteine, vitamins (1× MEM), ITS (1×), glucose (up to a total of 6 mM), 1.0 IU/ml equine chorionic gonadotropin (eCG), 2.0 IU/ml human chrorionic gonadotropin (hCG), 25 ng/ml epidermal growth factor (EGF) and 4 mg/ml bovine serum albumin (BSA), for 24 h. The cumulus cells were then removed using 0.3 mg/ml hyaluronidase.

### Ca^2+ ^imaging and fluorescence measurements

Mature oocytes were loaded with the Ca^2+ ^indicator dye fura-2 by incubation in the presence of 2 μM fura-2 AM (acetoxymethyl ester form) and 0.02% detergent pluronic F-127 (Invitrogen; Carlsbad, CA, U.S.A.) for 45 min. The oocytes were then rinsed and transferred to a chamber with a glass coverslip as the bottom. They were exposed to various treatments and changes in the [Ca^2+^]_i _were recorded using InCyt Im2, a dual-wavelength fluorescence imaging system (Intracellular Imaging, Inc.; Cincinnati, OH, U.S.A.). Fluorescence was recorded by calculating the ratio of fura-2 fluorescence at 510 nm excited by UV light alternatively at 340 and 380 nm. The Ca^2+ ^concentration was calculated by comparing the ratio of fluorescence at 340 and 380 nm against a standard curve of known Ca^2+ ^concentration prepared with fura-2 potassium salt (Invitrogen). During measurements, the baseline [Ca^2+^]_i _was detected first and then different stimuli were applied. For each treatment, the fluorescence measurement was performed for at least 10 min and then it was repeated several times using different oocytes.

### Oocyte treatments

A number of stimuli were tested for their ability to trigger an elevation in the [Ca^2+^]_i _of the oocytes. Then, based on the results of these measurements and on data published in other species, a number of treatments were identified that were deemed to have the potential to trigger parthenogenetic development of the oocytes. The oocytes were treated using the selected methods, then a portion of them were incubated in the presence of 10 μg/ml cycloheximide (a protein synthesis blocker) and 7.5 μg/ml cytochalasin B (an inhibitor of actin polymerization) for 4 h prior to culture; the rest of the oocytes were cultured without inhibitor supplementation. In previous studies the use of such inhibitors had a positive effect on embryo development following oocyte activation (for review see [21]. The oocytes were then cultured as described below and embryonic development induced by the different methods was monitored. The following treatments were tested.

#### Electric DC pulse

Individual oocytes were transferred to a chamber with two stainless steel electrodes where the bottom of the chamber had been replaced with a glass coverslip. The chamber was filled with electroporation medium (consisting of mannitol 0.3 M, CaCl_2 _0.1 mM, MgSO_4 _0.1 mM, HEPES 0.5 mM and BSA 0.01 mg/ml) and placed on the stage of an inverted microscope. After a 5-min equilibration period the steady-state Ca^2+ ^levels were measured, then two DC pulses of various characteristics (1.0 kV/cm for 20 μsec; 1.2 kV/cm for 60 μsec; or 2 kV/cm for 60 μsec), 1 sec apart, were applied to the oocytes [21] and changes in the [Ca^2+^]_i _were monitored. To stimulate development two DC pulses of either 1.0 kV/cm for 20 μsec or 1.2 kV/cm for 60 μsec were applied.

#### Ethanol

Oocytes were placed into the Ca^2+ ^measurement chamber containing TL-Hepes and ethanol (200 proof) was added to the medium to obtain 7% (v/v) final ethanol concentration [22]. Changes in the [Ca^2+^]_i _were recorded by the fluorescence imaging system. In order to trigger parthenogenetic development, oocytes were exposed to 7% (v/v) ethanol for 5 min.

#### Ionomycin

Each oocyte was transferred into TL-Hepes and ionomycin dissolved in dimethyl sulfoxide (DMSO) was added to the medium at a final concentration of 5 or 50 μM [23]. The same amount of DMSO without ionomycin was added to control oocytes. For parthenogenetic development, the oocytes were incubated in TL-Hepes in the presence of 5 or 50 μM ionomycin for 5 min.

#### Thimerosal

By oxidizing sulfhydryl groups on critical Ca^2+ ^release proteins thimerosal is known to elevate [Ca^2+^]_i _in oocytes [24]. To assess whether thimerosal has the same effect in cats, individual oocytes were transferred into TL-Hepes and thimerosal dissolved in TL-Hepes was added at a final concentration of 200 μM [25]. Because thimerosal oxidizes sulfhydryl groups on tubulin and thus damages the meiotic spindle, the application of a reducing agent such as dithiothreitol (DTT) is necessary to regenerate the spindle [26]. For activation, mature oocytes were treated according to two different schemes with the sulfhydryl modifying agents. In the first group, the oocytes were incubated in 200 μM thimerosal for 15 min followed by a 30-min incubation in the presence of 8 mM DTT. Oocytes in the other group were exposed to 200 μM thimerosal for 30 min followed by a 8 mM DTT treatment for 20 min.

#### Strontium

The oocytes were washed in Ca^2+^/Mg^2+^-free TL-Hepes medium and transferred to the measurement chamber containing 500 μl of the same medium. After recording the baseline Ca^2+ ^concentration, strontium-chloride (SrCl_2_) dissolved in TL-Hepes was added at a final concentration of 10 or 20 mM [27]. To induce development, oocytes were incubated in the presence of 10 or 20 mM SrCl_2 _for 4 h.

#### Na^+^-free medium

It was shown in pig oocytes that Na^+^-free medium can induce repetitive Ca^2+ ^transients via the Na^+^/Ca^2+ ^exchanger [28]. Based on this finding cat oocytes were placed individually into 10 μl TL-Hepes. Following baseline detection, 2 ml Na^+^-free medium was added to the medium that resulted in an approximately 200× dilution of the original Na^+ ^concentration. The Na^+^-free medium consisted of choline chloride 114 mM, KCl 3.2 mM, KH_2_PO_4 _0.4 mM, MgCl_2 _× 6H_2_O 0.5 mM, Hepes 9.2 mM, CaCl_2 _× 2H_2_O 2 mM, PVA 0.1%, pH 7.0. For activation, mature oocytes were incubated in Na^+^-free medium for 15, 30, 45 or 60 min.

### Embryo culture and evaluation

Potential zygotes were cultured (in groups of 10) in 100 μl drops of FOCM IVC-1 medium (FOCM supplemented with 0.4% BSA). To evaluate the formation of pronuclei the activated oocytes were cultured for 8 h, then they were mounted on microscope slides under posted coverslips and placed in a fixative of ethanol: acetic acid (3:1) for 7 days. They were then stained with 1% (w/v) aceto-orcein and evaluated for the presence or absence of pronuclei. In order to assess development to the blastocyst stage, after 3 days of culture the groups of embryos were transferred into 100 μl drops of FOCM IVC-2 medium (FOCM supplemented with 5% fetal bovine serum) and incubated for an additional 4 days [20]. At the end of the 7-day culture period, the embryos were incubated in 5 μg/ml Hoechst 33342 for 15 min and the nuclei showing blue fluorescence were counted using an epifluorescence microscope.

### Somatic cell nuclear transfer

#### Preparation of donor cells

The ultimate goal of these experiments was to generate cats as models for the heritable metabolic defect MPS I or AMD. The cell lines used as nuclear donors were generated by skin biopsies from affected cats. The explants were cut into small pieces and cultured in Dulbecco's Modified Eagle's medium (DMEM, Invitrogen) supplemented with 15% fetal bovine serum and 1% penicillin/streptomycin. Small aliquots of cells between passages 3 and 5 were frozen in DMEM supplemented with 10% DMSO and stored in liquid nitrogen until use.

#### Oocyte collection

Female cats were monitored daily for signs of behavioral estrus. Blood samples were collected from anestrual queens and the serum was assessed for progesterone to evaluate luteal status. Non-luteal queens were administered four intramuscular (i.m.) injections of porcine FSH at 24 h intervals (Day 1: 9.6 IU; Day 2: 7.6 IU; Day 3: 5.7 IU; Day 4: 5.7 IU); [29]. The last FSH injection was followed 24 h later by an i.m. injection of porcine LH (1,000 IU). Donors were anesthetized 24-27 h after the LH injection and subjected to laparoscopy and follicle aspiration. Follicles (2-5 mm in diameter) were aspirated and the COCs were collected in bicarbonate-buffered FOCM.

#### Nuclear transfer

Oocytes with an extruded polar body were enucleated in TL-Hepes medium supplemented with 7.5 μg/ml cytochalasin B. For enucleation, the first polar body and the adjacent cytoplasm presumably containing the metaphase-II chromosomes were removed. (Because in previous experiments this blind enucleation led to ~85% successfully enucleated oocytes, Hoechst staining was not used during nuclear transfer in the present study). A donor cell was then transferred into the perivitelline space of each enucleated oocyte. Reconstructed oocytes were placed in an electroporation chamber filled with Ca^2+^-free electroporation medium (containing 300 mM mannitol, 0.2 mM MgSO_4_, 0.5 mM Hepes, 0.1% polyvinyl alcohol) and two DC pulses (1.2 kV/cm for 60 μsec, 1 sec apart) were applied. Following fusion, part of the reconstructed oocytes were activated by electroporation (two pulses of 1.0 kV/cm for 20 μsec) and the rest by the combined method of 15 min incubation with 200 μM thimerosal followed by 30 min in 8 mM DTT. Treated oocytes were transferred to FOCM supplemented with 10 μg/ml cycloheximide and 7.5 μg/ml cytochalasin B for 4 h. Potential zygotes were then washed and cultured in FOCM/0.4% BSA until embryo transfer.

#### Embryo transfer

Estrual females, on Day 2 to 6 of estrus received two subcutaneous injections of gonadotropin releasing hormone (GnRH; 25 μg each, 12 h interval). The cats were anesthetized 54 to 58 h after the first GnRH injection and the ovaries were evaluated via laparoscopy. If fresh corpora lutea were present on the ovaries confirming the occurrence of recent ovulations, early cleavage stage (20-38 h after nuclear transfer) embryos (n = 8-21 per animal) were transferred through the ostium into the lumen of the left or right oviduct. All embryo recipient queens were subjected to ultrasound examination 21-23 days after embryo transfer. Pregnant females were reassessed with ultrasonography periodically to monitor fetal viability and growth.

### Statistical analysis

Frequencies of pronuclear formation, cleavage and blastocyst development were compared by the chi-square test. Total cell numbers of the blastocysts were compared by the Student's t-test. Statistical comparisons were performed using either SAS or Minitab; differences were considered significant at P < 0.05.

## Results

### Changes in cytosolic Ca^2+ ^levels

**Figure 1:**
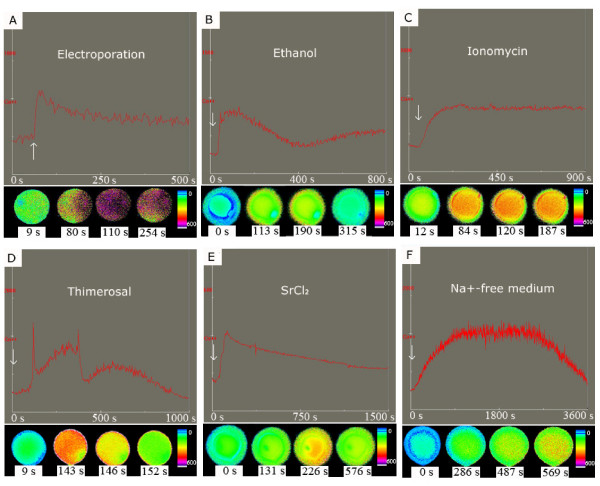
**Changes in the [Ca^2+ ^]_i _of cat oocytes induced by different stimuli**. The upper part of each section shows cytoplasmic free Ca^2+ ^levels as detected by a photometer; the arrows indicate the time when the stimulus was applied. In the lower part of each section images of [Ca^2+ ^]_i _changes in individual cat oocytes are shown; different colors indicate different intracellular free Ca^2+ ^concentrations.

Electroporation triggered an almost immediate rise in the [Ca^2+^]_i _of cat oocytes. The application of DC pulses of larger voltage or longer duration led to more robust changes in cytoplasmic Ca^2+ ^levels and increasing these parameters above a certain threshold (e.g. raising voltage above ~1.8 kV/cm) caused elevated intracellular Ca^2+ ^levels that did not return to baseline possibly indicating permanent membrane damage (data not shown). A typical change induced by two pulses of 1 kV/cm, 20 μsec each is shown in Figure 1A. The resting [Ca^2+^]_i _in the oocytes was approximately 200 nM. The [Ca^2+^]_i _elevation started 3 ± 0.4 sec after applying the electrical shock and peaked at a concentration of 485 ± 28 nM after which it decreased gradually. The transient had an average duration of 73 ± 15 sec and the [Ca^2+^]_i _reached near baseline levels by the end of the measurements (n = 15). Electroporation caused a single [Ca^2+^]_i _rise in all oocytes tested (Table 1).

**Table 1:** Characteristics of [Ca^2+ ^]_i _changes (mean ± SEM) induced by various artificial stimuli

Treatment	No. of oocytes (No. of responses)	Time till response (sec)	Peak [Ca^2+ ^]_i_ (nM)	Duration (sec)	Oscillation	Interval between peaks (sec)
Electroporation	15 (15)	3 ± 0.4	485 ± 28	73 ± 15	No	--
7% ethanol	5 (12)	110 ± 31	394 ± 87	150 ± 41	No	--
5 μM ionomycin	5 (5)	18 ± 2	277 ± 27	>800	No	--
50 μM ionomycin	5 (5)	6 ± 1	336 ± 12	>800	No	--
200 μM thimerosal	15 (18)	365 ± 51	496 ± 75	85 ± 8	Yes	351 ± 30
20 mM SrCl_2_	15 (15)	85 ± 10	431 ± 52	335 ± 28	No	--
Na^+^-free medium	15 (28)	135 ± 24	639 ± 79	1470 ± 242	No	--

Incubation of mature cat oocytes in 7% ethanol induced a single [Ca^2+^]_i _rise in 5 out of 12 oocytes (Figure 1B). The elevation started at 110 ± 11 sec after adding the ethanol; the [Ca^2+^]_i _then increased up to an average of 394 ± 87 nM and then returned to basal levels. The mean total duration of the Ca^2+ ^transient was 150 ± 41 sec. Ionomycin at both concentrations (5 and 50 μM) could elicit a rapid Ca^2+ ^response in all (a total of 10) oocytes tested. Figure 1C shows a [Ca^2+^]_i _change induced by 50 μM ionomycin. The [Ca^2+^]_i _rise in these cases started within a few seconds after adding ionomycin and peaked at a concentration of 336 ± 12 nM. In all cases the [Ca^2+^]_i _elevation had an extended duration that lasted as long as the oocyte was exposed to the ionophore since no [Ca^2+^]_i _return to basal levels was observed during the measurement period. The Ca^2+ ^rise triggered by 50 μM ionomycin started within a shorter period of time and had higher amplitude compared to those stimulated by lower ionomycin concentration (data not shown). DMSO alone did not elicit changes in the [Ca^2+^]_i _(data not shown).

Incubation of mature cat oocytes with 200 μM thimerosal induced [Ca^2+^]_i _elevation in 15 out of 18 oocytes; the signal took place as an oscillation in the intracytoplasmic Ca^2+ ^level in 11 oocytes. On average, the initial [Ca^2+^]_i _rise started 356 ± 51 sec after the addition of thimerosal, with an average peak concentration of 496 ± 75 nM and a mean duration of 85 ± 8 sec. Subsequent rises in the [Ca^2+^]_i _were generated at intervals of 351 ± 30 sec; the number of Ca^2+ ^spikes induced in the oocytes was between 2 and 5. In all oocytes the initial transient had the highest peak, each subsequent transient tended to have a lower average amplitude (Figure 1D). The oscillations usually lasted for 30-45 min and during this time the Ca^2+ ^baseline increased gradually.

Addition of SrCl_2 _caused a single long-lasting rise in the [Ca^2+^]_i _in all oocytes tested (n = 30). As a result of 20 mM SrCl_2 _stimulation, the baseline [Ca^2+^]_i _increased to 431 ± 52 nM within 85 ± 10 sec. The average duration of the elevated [Ca^2+^]_i _was 335 ± 28 sec (Figure 1E). The lower (10 mM) SrCl_2 _concentration elicited a similar response in the [Ca^2+^]_i _(data not shown).

The profile of the [Ca^2+^]_i _rise caused by Na^+^-free-medium is displayed in Figure 1F. Incubation in Na^+^-free-medium induced a gradual [Ca^2+^]_i _elevation in 15 out of 28 oocytes. The rise started 135 ± 24 sec after addition of the Na^+^-free-medium and peaked at a concentration of 639 ± 79 nM. The average duration of the [Ca^2+^]_i _rise induced by the Na^+^-free-medium was 1,470 ± 242 sec.

### Parthenogentic embryo development

The fluorescence recordings indicated that all the artificial stimuli tested were able to generate an elevation in the [Ca^2+^]_i _in mature cat oocytes. Next, in preliminary experiments the different stimuli were evaluated for their ability to induce parthenogenetic development and based on the outcome of these experiments four different treatments were finally selected and compared. The treatments selected for further evaluation were the following: 1) electroporation using two DC pulses of 1.0 kV/cm for 20 μsec each; 2) 15 min incubation in 200 μM thimerosal followed by a culture in 8 mM DTT for 30 min; 3) incubation in the presence of 20 mM SrCl_2 _in Ca^2+^/Mg^2+^-free medium for 4 h, and 4) a treatment in Na^+^-free medium for 45 min. Each treatment was applied with or without subsequent incubation in the presence of 10 μg/ml cycloheximide and 7.5 μg/ml cytochalasin B for 4 h.

**Figure 2:**
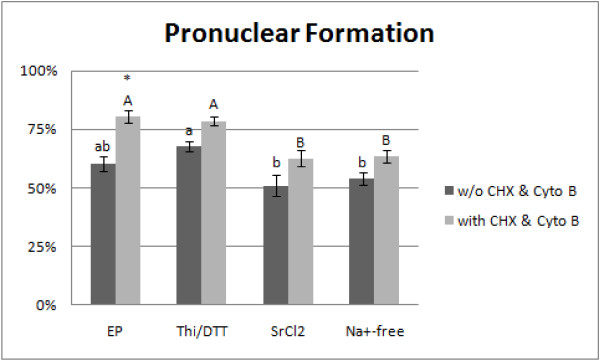
**Pronuclear formation in cat oocytes activated by electroporation (EP), thimerosal/DTT (Thi/DTT), SrCl_2 _and Na^+^-free medium (Na^+^-free) with or without 4-h incubation in cycloheximide (CHX) and cytochalasin B (Cyto B)**. The asterisk indicates a significant difference (P < 0.05) between frequencies of pronuclear formation achieved with and without CHX/Cyto B supplementation. Different lowercase letters indicate significant differences between treatments without CHX/Cyto B supplementation (P < 0.05); different uppercase letters indicate significant differences between treatments that were followed by CHX/Cyto B incubation (P < 0.05).

Electroportation stimulated 72 out of 119 (60.5 ± 2.9%) oocytes to form pronuclei. Incubation of the electroporated oocytes with cycloheximide and cytochalasin B increased the percentage of oocytes having pronuclei significantly (65 out of 81, 80.2 ± 3.1%, P < 0.05). The combined thimerosal/DTT treatment induced pronuclear formation in 84 out of 124 oocytes (67.7 ± 1.8%), this frequency was significantly higher than those found after the 20 mM SrCl_2 _(58/114; 50.9 ± 4.0%) and Na^+^-free medium (62/115; 53.9 ± 2.4%) treatments (P < 0.05). Oocytes in these groups tended to show higher pronuclear formation after cycloheximide and cytochalasin B incubation but the difference was not statistically significant (Figure 2).

**Figure 3:**
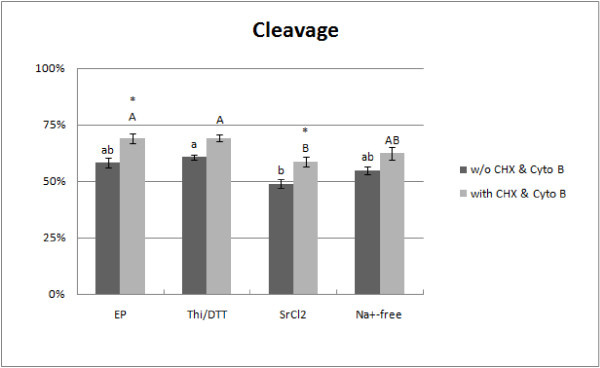
**Cleavage development of embryos after activation by electroporation (EP), thimerosal/DTT (Thi/DTT), SrCl_2 _and Na^+^-free medium (Na^+^-free) with or without a 4-h incubation in cycloheximide (CHX) and cytochalasin B (Cyto B)**. The asterisks indicate significant differences (P < 0.05) between cleavage frequencies achieved with and without CHX/Cyto B supplementation. Different lowercase letters indicate significant differences between treatments without CHX/Cyto B supplementation (P < 0.05); different uppercase letters indicate significant differences between treatments where the Ca^2+ ^signal-inducing stimulus was followed by CHX/Cyto B incubation (P < 0.05).

The cleavage frequency stimulated by electroporation was 58.2 ± 4.0% (142 out of 244 oocytes; Figure 3). The thimerosal/DTT treatment triggered 60.6 ± 2.4% (154/254) of the oocytes to cleave, this percentage was significantly higher than that registered in the SrCl_2_-treated group (128/262; 48.9 ± 3.6%; P < 0.05). Of the oocytes that were activated with Na^+^-free medium, 54.9 ± 3.4% (162/295) cleaved. We also found that incubation with cycloheximide and cytochalasin B caused a significant increase in cleavage frequency after electroporation (140/203; 69 ± 4.2%) and SrCl_2 _(118/201; 58.7 ± 4.5%) treatments (P < 0.05).

**Figure 4:**
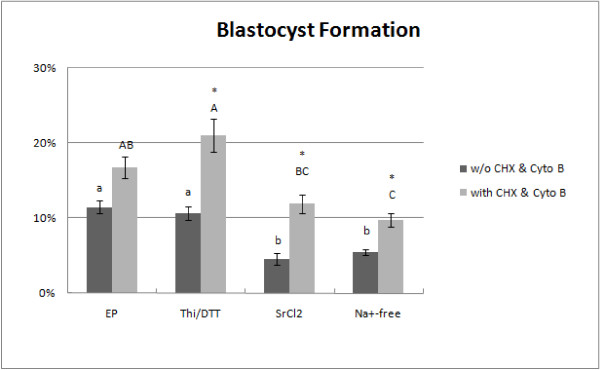
**Blastocyst formation induced by parthenogenetic activation of cat oocytes**. The asterisks indicate significant differences (P < 0.05) between frequencies of blastocyst formation achieved with and without CHX/Cyto B supplementation. Different lowercase letters indicate significant differences between treatments without CHX/Cyto B supplementation; different uppercase letters indicate significant differences between treatments followed by CHX/Cyto B incubation (P < 0.05).

In the absence of incubation with the inhibitors, blastocyst formation was the highest after electroporation (28/244; 11.5 ± 1.7%) and the combined thimerosal/DTT treatment (27/254; 10.6 ± 1.9%). These values were significantly higher than those in the SrCl_2 _(12/262; 4.6 ± 3.6%) and Na^+^-free medium (16/295; 5.4 ± 3.4%) - treated groups (P < 0.05; Figure 4). Incubation with cycloheximide and cytochalasin B significantly improved the frequency of blastocyst formation in oocytes that were activated by thimerosal/DTT (44/209; 16.7 ± 2.9%), SrCl_2 _(24/201; 11.9 ± 2.8%) and Na^+^-free medium (24/245; 9.8 ± 1.4%); (P < 0.05).

### Somatic cell nuclear transfer

A total of 157 reconstructed oocytes were produced by somatic cell nuclear transfer using MPS I-affected donor cells; these oocytes were activated by electroporation followed by incubation in cycloheximide and cytochalasin B. Following activation 114 (72.6%) reconstructed oocytes cleaved, the 2 to 4-cell embryos were transferred into the oviducts of 9 recipient females. Three of these recipients had implantations on initial palpation and ultrasonographic exam on Day 20 to 21 post-transfer. During early ultrasonography a total of 4 implantation sites and 3 developing fetuses were found; one female had only a single implantation site with no visible fetus. Heartbeats were successfully detected in two of the fetuses. However, by day 47 all 3 fetuses were absorbed (Table 2). An additional 130 reconstructed oocytes were produced by nuclear transfer using AMD-affected donor cells; 73 of which were activated by electroporation followed by cycloheximide and cytochalasin B incubation while 57 were activated by thimerosal/DTT followed by incubation with the inhibitors. In the electroporation-activated group 62 oocytes (84.9%) cleaved; cleavage frequency in the thimerosal/DTT-treated group was 77.2% (44 out of 57 oocytes). The 62 cleaved embryos that were produced by electroporation were transferred into 6 recipients, of which 2 became pregnant. In the pregnant animals 4 implantation sites and 2 fetuses were detected on day 21. The 2 to 4-cell embryos that resulted from the thimerosal/DTT activation were transferred into 4 recipients; ultrasonography on day 21 could find neither implantation sites nor developing fetuses in these surrogates. No fetuses could be detected by ultrasonography on day 49 in any of the recipient animals.

**Table 2:** Development of reconstructed cat oocytes activated by electroporation or thimerosal/DTT.

Donor Cell type	Activation treatment	Embryos produced	Embryos cleaved and transferred (%)	Recipients	Pregnant recipients	Implantation sites (%)	No. of fetuses (%)
MPS	Electroporation	157	114 (72.6)	9	2*	4 (3.5)	3 (2.6)
AMD	Electroporation	73	62 (84.9)	6	2	4 (6.5)	2 (3.2)
AMD	Thimerosal/DTT	57	44 (77.2)	4	0	0 (0)	0 (0)

Stimulated oocytes in both cases were subsequently incubated in cycloheximide and cytochalasin B for 4 h.

*A third female had a single implantation site but with no observable fetus

## Discussion

Following sperm-oocyte fusion, the Ca^2+ ^signal triggered by the fertilizing sperm is responsible for stimulating meiotic resumption and eventually, embryonic development. In most cases, parthenogenetic activation methods also induce an elevation in the [Ca^2+^]_i _to activate the oocyte's developmental program [5]. In many species numerous comparative studies have been carried out using different methods of artificial activation, and the most effective ones have been selected to activate reconstructed oocytes after nuclear transfer. In felids no such studies have been performed and the number of methods available for parthenogenetic activation of cat oocytes is rather limited. In the present study we characterized responses of cat oocytes to several artificial stimuli in order to find ways to trigger [Ca^2+^]_i _changes similar to those detected during mammalian fertilization. All the methods tested could elicit a rise in the [Ca^2+^]_i _of cat oocytes. Electroporation is commonly used to induce a transient elevation in the [Ca^2+^]_i _of oocytes of various species; it has also been successfully used to stimulate oocyte activation during cat nuclear transfer [30]. The short, high voltage DC pulses are known to induce a significant transmembrane Ca^2+ ^influx by causing a destabilization of the phospholipid bilayer. The influx is influenced by the voltage and duration of the electrical pulse as well as the ionic strength of the electroporation medium [31]. We evaluated the effect of DC pulses with various duration and magnetic field characteristics; all pulses triggered changes in the [Ca^2+^]_i_. To stimulate embryonic development we have selected a set of parameters that induced a large [Ca^2+^]_i _elevation without causing irreversible damage to the oocytes.

Ethanol was reported to activate oocytes of various species. In Xenopus it was demonstrated to stimulate the production of inositol 1,4,5-trisphosphate (IP_3_) which induces the release of Ca^2+ ^from the oocyte's intracellular stores [32]. Its effect on cytoplasmic Ca^2+ ^levels of cat oocytes has never been characterized before. We found that 7% ethanol triggered a long-lasting Ca^2+ ^signal in cat oocytes as well. However, the effects of ethanol on subsequent embryonic development have not been tested in the present study because recently it was shown by others that ethanol could activate ~50% of cat oocytes [8]. The Ca^2+ ^ionophore ionomycin has also been shown to induce [Ca^2+^]_i _elevations in occytes of many species. According to the literature, the parameters of ionomycin treatment used to activate oocytes of various species vary significantly: concentrations ranging from 5 μM to 5 mM [33] and treatment duration of 1 min to 40 min have also been reported [34]. The influence of ionomycin on cat oocytes has never been described; therefore optimizing the concentration and incubation time is critical for successful oocyte activation. As in other species, cat oocytes showed a larger elevation in the intracellular free Ca^2+ ^levels after being treated with higher concentrations of ionomycin. Surprisingly, all the ionomycin concentrations that we tested for the ability to induce [Ca^2+^]_i _changes caused irreversible damages and were lethal to the oocytes.

Thimerosal is known to oxidize sulfhydryl groups on intracellular Ca^2+ ^release proteins [35,36], thus causing the release of stored Ca^2+ ^and oscillation in the [Ca^2+^]_i _levels in oocytes of a number of species [24,26,37]. Similar to those findings, thimerosal in cat oocytes was able to induce repetitive [Ca^2+^]_i _changes. During incubation, the baseline [Ca^2+^]_i _became increasingly elevated between transients that was probably attributable to the inhibition of Ca^2+^-ATPases since it was reported that thimerosal not only induced Ca^2+ ^release from the stores but also inhibited Ca^2+^-ATPases and prevented the removal of excess Ca^2+ ^from the cytosol [38].

Incubation in the presence of Sr^2+ ^induces activation in mouse oocytes [39] but the mechanism of its action has not been completely elucidated. Because in certain cell types Sr^2+ ^is handled similar to Ca^2+ ^it was proposed that Sr^2+ ^activates oocytes by displacing bound Ca^2+ ^[40]. Recent studies showed that by using a low molecular weight heparin to antagonize the function of IP_3 _receptors or treating oocytes with the phospholipase C inhibitor U73122, the Sr^2+^-induced [Ca^2+^]_i _increases in mouse oocytes were blocked and this inhibitory effect could be rescued by microinjection of IP_3_. These results indicated that Sr^2+ ^triggered the release of stored Ca^2+ ^through the IP_3 _receptors [41]. Although in mice Sr^2+ ^treatment leads to an oscillation in the oocyte [Ca^2+^]_i_, we found that in cat oocytes Sr^2+ ^triggered a single, long-lasting Ca^2+ ^elevation.

The effects of Na^+^-free medium have been described in the pig where it was reported to induce oscillatory changes in the [Ca^2+^]_i_; [28]. In approximately 50% of the cases, Na^+^-free medium also stimulated a [Ca^2+^]_i _elevation in cat oocytes with a high amplitude and long duration. The effect of the Na^+^-free medium is probably mediated by the Na^+^/Ca^2+ ^exchanger expressed in the plasma membrane of cat oocytes. The Na^+^/Ca^2+ ^exchanger uses the energy of a Na^+ ^gradient to move Ca^2+ ^[42]. However, in case of low extracellular Na^+ ^levels the exchanger can operate in a reverse mode, pumping Ca^2+ ^into the cell. This feature of the exchanger makes Na^+^-free medium a potential candidate for stimulating oocyte activation. The exchanger was found to be present in hamster [43], mouse [44,45] and porcine [28] oocytes. Because Na^+^-free medium triggered an elevation in the [Ca^2+^]_i _we decided to investigate its ability to stimulate parthenogenetic development in cat oocytes.

Based on the results of the fluorescence measurements, four different methods have been selected to activate cat oocytes. As a result of the treatments, pronuclear formation in the oocytes ranged between 50.9% and 67.7%. The combined thimerosal/DTT treatment followed by cycloheximide and cytochalasin B incubation led to blastocyst formation in 21.1% of the cases. This frequency is similar to that received after electroporation, the method most widely used for oocyte activation. Although thimerosal is able to induce Ca^2+ ^oscillation in oocytes, it also oxidizes sulfhydryl groups on tubulin that prevents further development [46]. In order to eliminate its negative effects, the thimerosal treatment must be followed by an incubation in the presence of the sulfhydryl-reducing compound DTT [26]. The high percentage of blastocyst formation achieved in the present study indicates for the first time that the thimerosal/DTT activation is an effective way to stimulate parthenogenetic embryo development in cats.

Even though blastocyst formations induced by SrCl_2 _or Na^+^-free medium were somewhat low, this is the first report to demonstrate that these stimuli can trigger cat oocyte activation and subsequent blastocyst development. Although Na^+^-free medium has previously been reported to induce [Ca^2+^]_i _increases in porcine oocytes, no subsequent embryo development has been observed [28]. Here we found that Na^+^-free medium could induce cleavage of cat oocytes with a frequency similar to that triggered by electroporation or the combined thimerosal/DTT treatment, although the subsequent blastocyst formation was lower compared to the other treatments. It is possible that the amplitude of the Ca^2+ ^signal induced by the Na^+^-free medium is insufficient to induce complete egg activation. This is in accordance with the finding that in rabbit the amplitude of the Ca^2+ ^transient did not appear to affect early cleavage but it influenced the developmental competence of the produced embryo [7]. Another reason for the low blastocyst formation may be that sustained high levels of [Ca^2+^]_i _induced via the Na^+^/Ca^2+ ^exchanger caused cellular damage that negatively influenced developmental competence. Although optimization of the Na^+^-free treatment was attempted in this study, the improvement in embryonic development was not significant. Additional studies focusing on the fine-tuning of this method may be helpful to develop a method that utilizes the Ca^2+ ^signal generated via the Na^+^/Ca^2+ ^exchanger for oocyte activation.

The results obtained also showed that incubation of cat oocytes in cycloheximide and cytochalasin B after an induced Ca^2+ ^signal increased the frequency of pronuclear formation, cleavage and blastocyst formation. Mature mammalian oocytes arrested at the second metaphase stage synthesize cyclin B continuously in order to maintain activity of the M-phase Promoting Factor (MPF). During activation, MPF activity has to drop in order to allow release from the metaphase II arrest [47]. Cycloheximide is a protein synthesis inhibitor that blocks the production of cyclin B and in turn decreases MPF activity. Cytochalasin B on the other hand is an inhibitor of actin polymerization frequently used to block the extrusion of second polar body. In the presence of cytochalasin B, segregation of the chromosomes occurs after parthenogenetic oocyte activation but cytokinesis does not take place which results in the formation of diploid zygotes with two pronuclei [22,48]. In our experiments, incubation of cat oocytes with cycloheximide and cytochalasin B had the tendency to improve pronuclear formation, cleavage frequency and blastocyst development after every single Ca^2+ ^elevation-inducing stimulus tested and the improvement was significant in several cases. Although the incubation did not increase the total cell number of the developing blastocysts, the results indicate that these inhibitors are beneficial to increase the efficiency of oocyte activation in the cat.

The objective of the oocyte activation experiments was to develop potential methods to be used during nuclear transfer for the generation of useful disease models. In the domestic cat, somatic cell nuclear transfer not only provides the opportunity to generate genetically identical animals for research purposes, it also has the potential to facilitate preservation of rare and valuable felid populations, including cat research models and possibly endangered felid species [30]. In addition, domestic cats are useful research models for the study of more than 200 human hereditary diseases [49]. Maintenance of cat disease models can be challenging however, because the disease state may interfere with normal breeding, gestation and/or parturition. The application of nuclear transfer technology offers an innovative approach. Cloning would eliminate the need to collect gametes from affected animals for in vitro fertilization, it would obviate the requirement to maintain populations of carrier (heterozygous) cats, and for cat models that do not survive to sexual maturity cloning would provide a direct method for their continued propagation. Based on the frequency of blastocyst formation they induced, two methods were selected to activate reconstructed cat oocytes after nuclear transfer. These methods included electroporation and the combined thimerosal/DTT treatment, each followed by incubation in the presence of cycloheximide and cytochalasin B.

For nuclear transfer, fibroblast cells from two animals affected by different metabolic defects were collected and used as nuclear donors. The defects included AMD and MPS I, two lysosomal storage diseases that have been extensively characterized in cats. They are homologous to human inborn errors, involve single gene mutations and inherited as autosomal recessive traits [49,50]. These models have been instrumental in demonstrating the value of bone marrow transplantation in alleviating these diseases and, most recently, the potential of gene therapy using an adeno-associated viral vector for treating AMD-affected cats [14,15]. Cats affected with MPS I are difficult to breed naturally due to musculoskeletal abnormalities whereas AMD-affected cats typically die before reaching puberty.

Transfer of cloned embryos in which development was stimulated with electroporation followed by cycloheximide and cytochalasin B incubation resulted in 5 females having distinct implantation sites and 4 females with established pregnancies (i.e., presence of at least one fetus). Despite a total of 8 implantation sites, 5 fetuses and detectable heartbeats in two fetuses, no term development occurred; all fetuses were eventually reabsorbed by day 49 of pregnancy. Our previous research has demonstrated the developmental competence to term of MPS-affected and MPS-carrier IVF embryos following transfer into our primary recipient type [51]. Pregnancy loss in the current study may be attributable to deficient reprogramming of the donor nucleus in the enucleated oocyte or aberrant chromosome numbers as a result of incomplete enucleation. Furthermore, no pregnancies were detected following thimerosal/DTT-induced oocyte activation which was somewhat unexpected. Nevertheless, the thimerosal/DTT treatment stimulated cleavage divisions at a frequency comparable to that achieved by electroporation. This, in light of a recent report showing that the thimerosal/DTT activation supported term development of cloned transgenic piglets [52] implies that successful activation can be achieved by modification of sulfhydryl groups on key Ca^2+ ^releasing proteins in cat oocytes.

## Conclusion

This study described a number of novel methods to induce Ca^2+ ^release in cat oocytes for the stimulation of embryo development. In addition, the results support previous findings that electroporation is an effective way for the activation of oocytes reconstructed by nuclear transfer. Based on in vitro data, the combined thimerosal/DTT activation may also be suitable for cat oocyte activation but this needs further verification from additional studies.

## Competing interests

The authors declare that they have no competing interests.

## Authors' contributions

CW carried out in vitro oocyte maturation, fluorescence measurements, parthenogenetic oocyte activation, performed statistical analysis and drafted the manuscript. WFS conceived of the study, participated in its design and performed oocyte collections and embryo transfers. JRH participated in oocyte collections and embryo transfers. KL performed nuclear transfer. ZM participated in the design and coordination of the study and helped to draft the manuscript. All authors read and approved the final manuscript.

## References

[B1] Sagata N Meiotic metaphase arrest in animal oocytes: its mechanisms and biological significance Trends Cell Biol 1996 6 1 22 28 15157528 10.1016/0962-8924(96)81034-8

[B2] Cuellar O Animal parthenogenesis Science 1977 197 4306 837 843 887925 10.1126/science.887925

[B3] Whitaker M Larman MG Calcium and mitosis Semin Cell Dev Biol 2001 12 1 53 58 11162747 10.1006/scdb.2000.0217

[B4] Alberio R Zakhartchenko V Motlik J Wolf E Mammalian oocyte activation: lessons from the sperm and implications for nuclear transfer Int J Dev Biol 2001 45 7 797 809 11732839

[B5] Machaty Z Prather RS Strategies for activating nuclear transfer oocytes Reprod Fertil Dev 1998 10 7-8 599 613 10612466 10.1071/rd98048

[B6] Fulton BP Whittingham DG Activation of mammalian oocytes by intracellular injection of calcium Nature 1978 273 5658 149 151 565475 10.1038/273149a0

[B7] Ozil JP Huneau D Activation of rabbit oocytes: the impact of the Ca^2+ ^signal regime on development Development 2001 128 6 917 928 11222146 10.1242/dev.128.6.917

[B8] Grabiec A Max A Tischner M Parthenogenetic activation of domestic cat oocytes using ethanol, calcium ionophore, cycloheximide and a magnetic field Theriogenology 2007 67 4 795 800 17134746 10.1016/j.theriogenology.2006.10.009

[B9] Shelton GD Engvall E Canine and feline models of human inherited muscle diseases Neuromuscul Disord 2005 15 2 127 138 15694134 10.1016/j.nmd.2004.10.019

[B10] Dias AS Bester MJ Britz RF Apostolides Z Animal models used for the evaluation of antiretroviral therapies Curr HIV Res 2006 4 4 431 446 17073618 10.2174/157016206778560045

[B11] Coyne KP Edwards D Radford AD Cripps P Jones D Wood JL Gaskell RM Dawson S Longitudinal molecular epidemiological analysis of feline calicivirus infection in an animal shelter: a model for investigating calicivirus transmission within high-density, high-turnover populations J Clin Microbiol 2007 45 10 3239 3244 17687017 10.1128/JCM.01226-07 PMC2045375

[B12] Gillick M Linn K Rotating dome trochleoplasty: an experimental technique for correction of patellar luxation using a feline model Vet Comp Orthop Traumatol 2007 20 3 180 184 17846683 10.1160/vcot-06-09-0071

[B13] Wakeling J Smith K Scase T Kirkby R Elliott J Syme H Subclinical hyperthyroidism in cats: a spontaneous model of subclinical toxic nodular goiter in humans? Thyroid 2007 17 12 1201 1209 18177255 10.1089/thy.2007.0225

[B14] Ellinwood NM Vite CH Haskins ME Gene therapy for lysosomal storage diseases: the lessons and promise of animal models J Gene Med 2004 6 5 481 506 15133760 10.1002/jgm.581

[B15] Vite CH McGowan JC Niogi SN Passini MA Drobatz KJ Haskins ME Wolfe JH Effective gene therapy for an inherited CNS disease in a large animal model Ann Neurol 2005 57 3 355 364 15732095 10.1002/ana.20392

[B16] Luvoni GC Pellizzari P Embryo development in vitro of cat oocytes cryopreserved at different maturation stages Theriogenology 2000 53 8 1529 1540 10883841 10.1016/S0093-691X(00)00295-8

[B17] Gomez MC Jenkins JA Giraldo A Harris RF King A Dresser BL Pope CE Nuclear transfer of synchronized african wild cat somatic cells into enucleated domestic cat oocytes Biol Reprod 2003 69 3 1032 1041 12773426 10.1095/biolreprod.102.014449

[B18] Swanson WF Application of assisted reproduction for population management in felids: the potential and reality for conservation of small cats Theriogenology 2006 66 1 49 58 16650889 10.1016/j.theriogenology.2006.03.024

[B19] Gomez MC Pope CE Giraldo A Lyons LA Harris RF King AL Cole A Godke RA Dresser BL Birth of African Wildcat cloned kittens born from domestic cats Cloning Stem Cells 2004 6 3 247 258 15671671 10.1089/clo.2004.6.247

[B20] Herrick JR Bond JB Magarey GM Bateman HL Krisher RL Dunford SA Swanson WF Toward a feline-optimized culture medium: impact of ions, carbohydrates, essential amino acids, vitamins, and serum on development and metabolism of in vitro fertilization-derived feline embryos relative to embryos grown in vivo Biol Reprod 2007 76 5 858 870 17267698 10.1095/biolreprod.106.058065

[B21] Machaty Z Activation of oocytes after nuclear transfer Methods Mol Biol 2006 348 43 58 16988371 10.1007/978-1-59745-154-3_3

[B22] Presicce GA Yang X Parthenogenetic development of bovine oocytes matured in vitro for 24 hr and activated by ethanol and cycloheximide Mol Reprod Dev 1994 38 4 380 385 7980946 10.1002/mrd.1080380405

[B23] Heytens E Soleimani R Lierman S De Meester S Gerris J Dhont M Elst J Van der De Sutter P Effect of ionomycin on oocyte activation and embryo development in mouse Reprod Biomed Online 2008 17 6 764 771 19079959 10.1016/s1472-6483(10)60403-8

[B24] Swann K Thimerosal causes calcium oscillations and sensitizes calcium-induced calcium release in unfertilized hamster eggs FEBS Lett 1991 278 2 175 178 1991508 10.1016/0014-5793(91)80110-o

[B25] Im GS Seo JS Hwang IS Kim DH Kim SW Yang BC Yang BS Lai L Prather RS Development and apoptosis of pre-implantation porcine nuclear transfer embryos activated with different combination of chemicals Mol Reprod Dev 2006 73 9 1094 1101 16736528 10.1002/mrd.20455

[B26] Machaty Z Wang WH Day BN Prather RS Complete activation of porcine oocytes induced by the sulfhydryl reagent, thimerosal Biol Reprod 1997 57 5 1123 1127 9369179 10.1095/biolreprod57.5.1123

[B27] Kishigami S Wakayama T Efficient strontium-induced activation of mouse oocytes in standard culture media by chelating calcium J Reprod Dev 2007 53 6 1207 1215 17938555 10.1262/jrd.19067

[B28] Machaty Z Ramsoondar JJ Bonk AJ Prather RS Bondioli KR Na^+^/Ca^2+ ^exchanger in porcine oocytes Biol Reprod 2002 67 4 1133 1139 12297528 10.1095/biolreprod67.4.1133

[B29] Pope CE In vitro fertilization and embryo transfer in felids Methods Mol Biol 2004 254 227 244 15041765 10.1385/1-59259-741-6:227

[B30] Gomez MC Pope CE Dresser BL Nuclear transfer in cats and its application Theriogenology 2006 66 1 72 81 16620927 10.1016/j.theriogenology.2006.03.017

[B31] Serpersu EH Kinosita K Jr Tsong TY Reversible and irreversible modification of erythrocyte membrane permeability by electric field Biochim Biophys Acta 1985 812 3 779 785 3970906 10.1016/0005-2736(85)90272-x

[B32] Ilyin V Parker I Effects of alcohols on responses evoked by inositol trisphosphate in Xenopus oocytes J Physiol 1992 448 339 354 1375639 10.1113/jphysiol.1992.sp019045 PMC1176203

[B33] Meo SC Yamazaki W Ferreira CR Perecin F Saraiva NZ Leal CL Garcia JM Parthenogenetic activation of bovine oocytes using single and combined strontium, ionomycin and 6-dimethylaminopurine treatments Zygote 2007 15 4 295 306 17967209 10.1017/S0967199407004285

[B34] Lan GC Han D Wu YG Han ZB Ma SF Liu XY Chang CL Tan JH Effects of duration, concentration, and timing of ionomycin and 6-dimethylaminopurine (6-DMAP) treatment on activation of goat oocytes Mol Reprod Dev 2005 71 3 380 388 15806561 10.1002/mrd.20267

[B35] Abramson JJ Cronin JR Salama G Oxidation induced by phthalocyanine dyes causes rapid calcium release from sarcoplasmic reticulum vesicles Arch Biochem Biophys 1988 263 2 245 255 2454077 10.1016/0003-9861(88)90633-9

[B36] Trimm JL Salama G Abramson JJ Sulfhydryl oxidation induces rapid calcium release from sarcoplasmic reticulum vesicles J Biol Chem 1986 261 34 16092 16098 3782109

[B37] Fissore RA Robl JM Sperm, inositol trisphosphate, and thimerosal-induced intracellular Ca^2+ ^elevations in rabbit eggs Dev Biol 1993 159 1 122 130 8365556 10.1006/dbio.1993.1226

[B38] Sayers LG Brown GR Michell RH Michelangeli F The effects of thimerosal on calcium uptake and inositol 1,4,5-trisphosphate-induced calcium release in cerebellar microsomes Biochem J 1993 289 Pt 3 883 887 8435083 10.1042/bj2890883 PMC1132258

[B39] Wakayama T Perry AC Zuccotti M Johnson KR Yanagimachi R Full-term development of mice from enucleated oocytes injected with cumulus cell nuclei Nature 1998 394 6691 369 374 9690471 10.1038/28615

[B40] Whittingham DG Siracusa G The involvement of calcium in the activation of mammalian oocytes Exp Cell Res 1978 113 2 311 317 299649 10.1016/0014-4827(78)90371-3

[B41] Zhang D Pan L Yang LH He XK Huang XY Sun FZ Strontium promotes calcium oscillations in mouse meiotic oocytes and early embryos through InsP_3 _receptors, and requires activation of phospholipase and the synergistic action of InsP_3_ Hum Reprod 2005 20 11 3053 3061 16055456 10.1093/humrep/dei215

[B42] Nicoll DA Longoni S Philipson KD Molecular cloning and functional expression of the cardiac sarcolemmal Na^+^-Ca^2+ ^exchanger Science 1990 250 4980 562 565 1700476 10.1126/science.1700476

[B43] Igusa Y Miyazaki S Effects of altered extracellular and intracellular calcium concentration on hyperpolarizing responses of the hamster egg J Physiol 1983 340 611 632 6887062 10.1113/jphysiol.1983.sp014783 PMC1199230

[B44] Carroll J Na^+^-Ca^2+ ^exchange in mouse oocytes: modifications in the regulation of intracellular free Ca^2+ ^during oocyte maturation J Reprod Fertil 2000 118 2 337 342 10864798 10.1530/jrf.0.1180337

[B45] Pepperell JR Kommineni K Buradagunta S Smith PJ Keefe DL Transmembrane regulation of intracellular calcium by a plasma membrane sodium/calcium exchanger in mouse ova Biol Reprod 1999 60 5 1137 1143 10208975 10.1095/biolreprod60.5.1137

[B46] Kuriyama R Sakai H Role of tubulin-SH groups in polymerization to microtubules. Functional-SH groups in tubulin for polymerization J Biochem 1974 76 3 651 654 4474165 10.1093/oxfordjournals.jbchem.a130609

[B47] Hashimoto N Watanabe N Furuta Y Tamemoto H Sagata N Yokoyama M Okazaki K Nagayoshi M Takeda N Ikawa Y Aizawai S Parthenogenetic activation of oocytes in c-mos-deficient mice Nature 1994 370 6484 68 71 8015610 10.1038/370068a0

[B48] Liu L Ju JC Yang X Parthenogenetic development and protein patterns of newly matured bovine oocytes after chemical activation Mol Reprod Dev 1998 49 3 298 307 9491382 10.1002/(SICI)1098-2795(199803)49:3<298::AID-MRD10>3.0.CO;2-T

[B49] O'Brien SJ Menotti-Raymond M Murphy WJ Yuhki N The Feline Genome Project Annu Rev Genet 2002 36 657 686 12359739 10.1146/annurev.genet.36.060602.145553

[B50] Winchester B Vellodi A Young E The molecular basis of lysosomal storage diseases and their treatment Biochem Soc Trans 2000 28 2 150 154 10816117 10.1042/bst0280150

[B51] Swanson WF Research in nondomestic species: experiences in reproductive physiology research for conservation of endangered felids ILAR J 2003 44 4 307 316 13130161 10.1093/ilar.44.4.307 PMC7108633

[B52] Hao YH Yong HY Murphy CN Wax D Samuel M Rieke A Lai L Liu Z Durtschi DC Welbern VR Price EM McAllister RM Turk JR Laughlin MH Prather RS Rucker EB Production of endothelial nitric oxide synthase (eNOS) over-expressing piglets Transgenic Res 2006 15 6 739 750 17080303 10.1007/s11248-006-9020-8

